# Supplementary material for: A Novel Anti-CD73 Antibody That Selectively Inhibits Membrane CD73 Shows Antitumor Activity and Induces Tumor Immune Escape
Source: Biomedicines. 2022 Mar 31;10(4):825. doi: 10.3390/biomedicines10040825 (PMC9031174; doi:10.3390/biomedicines10040825)
Supplement: Supplementary file 1 [file biomedicines-10-00825-s001.zip › biomedicines-1638395-supplementary.pdf]

Supplementary information

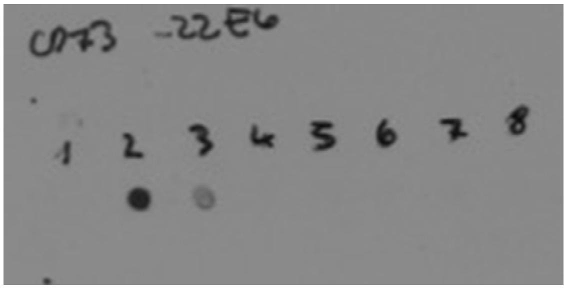

Figure S1: Characterization of EVs isolated from a malignant ascites. as described.

EVs precipitated by ultracentrifugation were floated into an Optiprep gradient, and fractions were tested for CD73

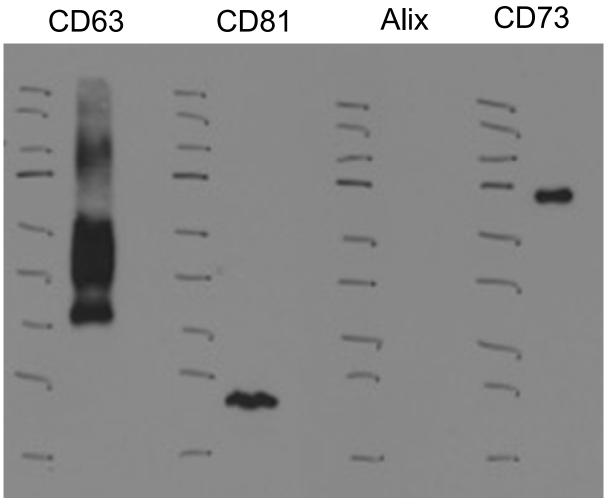

EVs from fraction 2 of the Optiprep gradient shown above were further characterized for the presence of CD63, CD81, Alix and CD73.

:

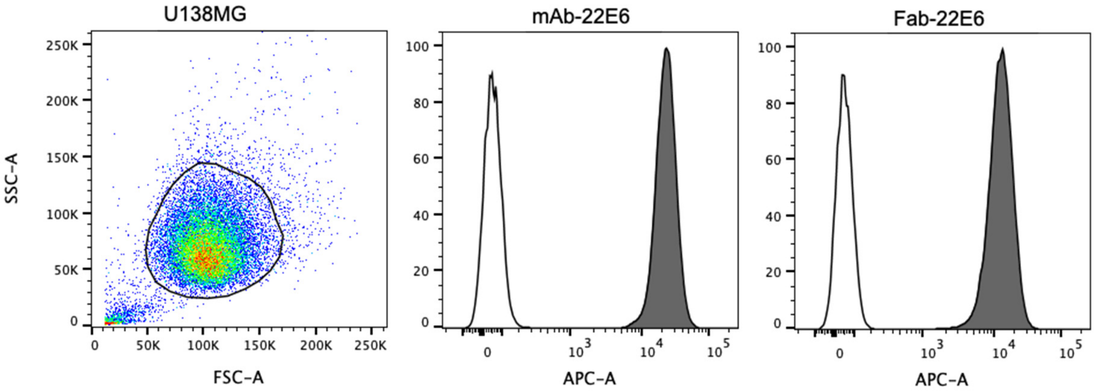

Figure S2. Binding of the antibody (mAb-22E6) and the Fab fragment (Fab-22E6) to U138MG cells. Uncolored histogram = isotype control.colored histogram = isotype control.
